# Real-world treatment patterns and overall survival among men with Metastatic Castration-Resistant Prostate Cancer (mCRPC) in the US Medicare population

**DOI:** 10.1038/s41391-023-00725-8

**Published:** 2023-10-02

**Authors:** Stephen J. Freedland, Matthew Davis, Andrew J. Epstein, Bhakti Arondekar, Jasmina I. Ivanova

**Affiliations:** 1 Department of Urology, Cedars-Sinai Medical Center, Los Angeles, CA USA; 2 Urology Section, Durham VA Medical Center, Durham, NC USA; 3 Medicus Economics, LLC, Milton, MA USA; 4 Pfizer Inc., Collegeville, PA USA; 5 Pfizer Inc., New York, NY USA

**Keywords:** Outcomes research, Prostate cancer

## Abstract

**Background:**

Real-world treatment patterns and survival in metastatic castration-resistant prostate cancer (mCRPC) have not been characterized for the full fee-for-service Medicare population.

**Methods:**

Men newly diagnosed with mCRPC were identified in Medicare fee-for-service claims during 1/1/2014–6/30/2019. Men had evidence of mCRPC and continuous insurance coverage ≥1 year before and ≥6 months after diagnosis unless patients died. Treatment patterns after diagnosis were described. Survival from mCRPC diagnosis and from start of first-line (1 L) therapy was modeled using Kaplan-Meier analysis.

**Results:**

Among 14,780 men with mCRPC, mean age was 76 and median follow-up after mCRPC was 17.0 months. 22% received no life-prolonging therapy after mCRPC, 78% received ≥1 line of therapy (LOT), 42% underwent ≥2 LOTs, and 20% had ≥3 LOTs. Median time from start of 1 L to next LOT or end of follow-up was 13.7 months, 10.9 months from 2 L start, and 8.9 months from 3 L start. The most common 1 L to 2 L treatment sequences among men with ≥2 lines were NHT followed by a different NHT (33%), chemotherapy followed by NHT (14%), and NHT followed by chemotherapy (13%). For those initiating 1 L treatment with NHTs, only 28% received subsequent treatment with a different class of therapy. Median survival was 25.6 months after mCRPC and 23.4 months following treatment initiation.

**Conclusions:**

More than 1 in 5 Medicare patients with mCRPC did not receive any life-prolonging therapy, and less than half received 2 L therapy. NHTs were the most common 1 L and 2 L therapies, with patients treated with NHT as 1 L followed by a different NHT for 2 L as the most common treatment sequence. Median survival from diagnosis for all patients was 25.6 months. These data highlight the dramatic undertreatment that occurs for mCRPC patients, particularly for therapies beyond NHTs as well as the common use of sequential NHTs in real-world data.

## Introduction

In the last decade, multiple life-prolonging therapies have been approved for the treatment of metastatic castration-resistant prostate cancer (mCRPC), increasing options available to patients and providers [1, 2]. These include chemotherapy (docetaxel and cabazitaxel), immunotherapy (sipuleucel-T), novel hormonal therapy (abiraterone and enzalutamide), poly (adenosine diphosphate-ribose) polymerase (PARP) inhibitors (olaparib and rucaparib), and radiopharmaceuticals (radium-223 and lutetium Lu 177 vipivotide tetraxetan). Given the multiple treatment options, it is crucial to understand, in real-world data, how the various treatments are used to identify gaps in treatments to optimize care of men with mCRPC. Toward this effort, recent studies evaluated mCRPC treatment patterns in electronic health records [1, 2] and commercial claims [3–9]. However, there are no studies focusing on treatment patterns and outcomes in the Medicare population. Patients aged 65 years and older represent 60% of incident prostate cancer cases [10], and Medicare is an important insurer in this age group [11].

This study undertook an evaluation of Medicare patients aged 65 years and older to characterize treatment patterns, including treatment sequencing and duration of therapy across lines of life-prolonging treatment, as well as survival outcomes.

## Materials and Methods

### Study sample

The study-eligible population included Medicare beneficiaries enrolled in Medicare Parts A, B, and D between January 1, 2014, and December 31, 2019. Using a modified version of the algorithm developed by Freedland et al. [12], 119,801 men were identified with metastatic prostate cancer. From this group, 41,927 men were identified with castration resistance as defined by documented medical castration-resistance, surgical castration-resistance, or medication used for castration-resistant mCRPC. An index date for each patient was assigned as the later of (a) the first claim with a diagnosis code in any position for metastatic disease occurring on or after the first claim with a diagnosis code in any position for prostate cancer, and (b) the first claim with evidence of castration resistance. Evidence of castration resistance was based on any of the following:

- ≥1 claim with a diagnosis code for hormone resistance (International Classification of Diseases, Tenth Revision, Clinical Modification [ICD-10-CM]: Z19.2);
- surgical castration at any time and ≥1 claim for rising prostate-specific antigen after (ICD-10-CM: R97.21);
- medication for mCRPC identifying castration resistance:
  i.) ≥1 claim for cabazitaxel, enzalutamide, mitoxantrone, radium-223, or sipuleucel-T;
  ii.) ≥1 claim for abiraterone acetate before June 2017 or initial claim for abiraterone acetate ≥90 days after initiation of androgen deprivation therapy (ADT); or
  iii.) initial claim for docetaxel ≥90 days after initiation of ADT;
- initial metastatic diagnosis occurring ≥90 days after surgical castration; or
- initial metastatic diagnosis occurring after 90 days of medical castration episode (ending within 30 days of or after initial metastatic diagnosis).

**Fig. 1:**
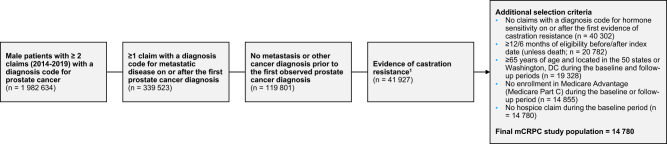
Sample selection. Evidence of castration resistance is based on any of the following: **a** ≥1 claim with a diagnosis code for hormone resistance (ICD-10-CM: Z19.2), **b** Surgical castration at any time AND ≥ 1 claim for rising prostate-specific antigen after (ICD-10-CM: R97.21), **c** Medication for mCRPC identifying castration resistance: (i) ≥1 claim for cabazitaxel, enzalutamide, mitoxantrone, radium-223, or sipuleucel-T; (ii) ≥1 claim for abiraterone acetate before June 2017 or initial claim for abiraterone acetate ≥90 days after initiation of ADT; or (iii) initial claim for docetaxel ≥90 days after initiation of ADT, **d** Initial metastatic diagnosis occurring ≥90 days after surgical castration, or **e** Initial metastatic diagnosis occurring after 90 days of medical castration episode (ending within 30 days of or after initial metastatic diagnosis).

Patients were required to have 12 or more months of eligibility prior to their index date and at least 6 months of eligibility following their index date unless patients died. Patients 65 years and older located in the 50 states and Washington DC were evaluated during the study period. To ensure full data capture, beneficiaries with any enrollment in Medicare Part C (Medicare Advantage) were excluded. Finally, considering their goals of care, patients enrolled in hospice prior to their incident mCRPC diagnosis were also excluded. The final study population consisted of 14,780 men (Fig. 1).

### Treatment type and line of therapy definitions

Patient characteristics at baseline were measured using claims from the 365-day period prior to the index date. Overall patient comorbidity burden was derived using the Charlson Comorbidity Index, modified to exclude prostate cancer given the study cohort [13]. The follow-up period lasted from the mCRPC index date until the end of data availability (December 31, 2019) or death.

Life-prolonging therapies during follow-up were grouped into 6 treatment categories of interest: (1) novel hormonal therapies (NHTs) defined as abiraterone, apalutamide, darolutamide, and enzalutamide; (2) chemotherapy defined as usage of cabazitaxel, docetaxel, carboplatin, cisplatin, oxaliplatin, mitoxantrone, and etoposide; (3) sipuleucel-T; (4) radium-223; (5) other treatments, including pembrolizumab, talazoparib, niraparib; and (6) combination therapy defined as any combined usage of treatment groups 1 through 5 within 28 days of the start of each line of therapy (LOT). Patients who did not receive any of the above listed therapies were categorized as untreated with life-prolonging therapy.

LOT was defined using the methodology described by George et al. and Shore et al. [1, 2]. The first LOT start date was defined as the date of the first claim for a life-prolonging treatment from the treatment categories described above up to 14 days prior to the index date. LOTs included all life-prolonging treatments initiated within 28 days of the LOT start date and ended with a treatment change or at least 90 days without treatment.

Time on each LOT was measured by calculating the duration of 1 L, 2 L, and 3 L treatment from the start of each LOT to the end of last treatment or initiation of a subsequent LOT. Time to next treatment was measured as the time from the initiation of a LOT to the initiation of the next LOT or end of follow-up observation. Thus, a patient spending 4 months on 1 L, pausing for 2 months, and then beginning 2 L would be characterized as having 4 months on 1 L and 6 months to 2 L.

### Statistical analysis

Baseline characteristics across the treatment groups outlined above were evaluated using χ^2^ and Wilcoxon rank sum tests for categorical and continuous measures, respectively, with NHT as the reference group because NHT was the most common 1 L treatment after mCRPC diagnosis.

To characterize contemporary real-world treatment patterns, the frequency and proportion of patients receiving different LOTs, regimens in each LOT, and regimen sequences across LOTs were described. Descriptive statistics were calculated for the duration of the overall study period (from index date to end of follow-up), duration of 1 L, 2 L, 3 L periods, and duration of 1 L, 2 L, 3 L treatments. Descriptive statistics and Kaplan-Meier curves of time from mCRPC index date to the start of 1 L treatment, from the end of 1 L to the start of 2 L, and from the end of 2 L to the start of 3 L were calculated.

Kaplan-Meier estimates for overall survival, defined as the time from mCRPC index date to the date of death or censoring for end of follow-up, was calculated. Similarly, survival time from treatment start, defined as the time from 1 L initiation to death or loss to follow-up, was also estimated for the subgroup of patients with life-prolonging treatment after mCRPC diagnosis.

Statistical analyses were performed using SAS software v9.4 (SAS Institute, Cary, NC). The study protocol was reviewed by the WIRB-Copernicus Group® (Princeton, NJ) institutional review board (IRB) and determined to be exempt from IRB review.

## Results

### Sample characteristics

The 14,780 men with mCRPC were 76 years of age on average at the time of mCRPC diagnosis (Table 1; Supplemental Table S1). The majority (75%) of patients were White, 14% were Black, and 6% Hispanic. As of the index date, 36%, 26%, and 20% of patients resided in the South, Midwest, and West, respectively, with the remaining 18% in the Northeast. Ten per cent of patients had undergone NHT use prior to their mCRPC diagnosis, and 3% had previously been treated with taxane-based chemotherapy.

**Table 1:** Baseline characteristics by 1 L treatment category^1,2^.

Characteristic	Overall	NHT	Chemotherapy	Sipuleucel-T	Radium-223	Combination & Other	Without life-prolonging treatment
N (%)	14780 (100%)	7556 (51%)	2005 (14%)	1189 (8%)	317 (2%)	461 (3%)	3252 (22%)
**Demographics**							
**Age in years, mean (SD)**	76.1 (7.3)	76.7 (7.3)	72.7* (5.0)	73.7* (6.1)	75.8 (7.0)	74.5* (6.4)	78.1* (7.9)
**Race, n (%)**							
Non-Hispanic White	11033 (75%)	5494 (73%)	1553 (77%*)	959 (81%*)	266 (84%*)	368 (80%*)	2393 (74%)
Black or African American	2079 (14%)	1117 (15%)	244 (12%*)	132 (11%*)	25 (8%*)	48 (10%*)	513 (16%)
Asian/Pacific Islander	381 (3%)	221 (3%)	44 (2%)	18 (2%*)	(redacted)	12 (3%)	79 (2%)
Hispanic	824 (6%)	479 (6%)	88 (4%*)	43 (4%*)	(redacted)	23 (5%)	181 (6%)
Other/Unknown^3^	463 (3%)	245 (3%)	76 (4%)	37 (3%)	(redacted)	(redacted)	86 (3%)
**Geographic region, n (%)**							
Northeast	2709 (18%)	1446 (19%)	338 (17%)	195 (16%)	58 (18%)	82 (18%)	590 (18%)
Midwest	3857 (26%)	1884 (25%)	571 (28%*)	311 (26%)	91 (29%)	106 (23%)	894 (27%*)
South	5287 (36%)	2607 (35%)	710 (35%)	463 (39%*)	106 (33%)	172 (37%)	1229 (38%*)
West	2927 (20%)	1619 (21%)	386 (19%)	220 (19%)	62 (20%)	101 (22%)	539 (17%*)
**Baseline treatment**							
Baseline NHT use	1519 (10%)	1060 (14%)	111 (6%*)	30 (3%*)	21 (7%*)	97 (21%*)	200 (6%*)
Baseline taxane use	461 (3%)	31 (0%)	366 (18%*)	(redacted)	(redacted)	23 (5%*)	33 (1%*)
**CCI, mean (SD)**	2.1 (2.0)	2.2 (2.1)	1.8* (1.8)	1.6* (1.7)	2.0 (1.8)	2.0 (2.0)	2.4* (2.2)

*P ≤ 0.01 vs NHT treatment; *CCI* Charlson Comorbidity Index, *NHT* Novel hormonal therapy, *SD* Standard deviation

1. Individuals are categorized into treatment categories based on their 1 L treatment.

2. Counts and proportions for cell sizes <11 are redacted.

3. Other/unknown includes 60% unknown, 30% other, and 11% American Indian/Alaska Native.

### mCRPC treatment

Overall, 78% of patients received one or more life-prolonging therapies following their diagnosis. Patients who did not receive any life-prolonging therapy were, on average, older and had a higher mean comorbidity burden compared to patients who had life-prolonging therapy (Table 1). Further, patients who received multiple lines of life-prolonging therapy were on average younger and had a lower mean comorbidity burden at baseline than those with only one line of treatment (Supplemental Table S2).

Among men receiving therapy, the most common 1 L treatment was NHT (66%), followed by chemotherapy (17%), sipuleucel-T (10%), combination & other therapy (4%), and finally radium-223 (3%). Patients receiving chemotherapy or sipuleucel-T were, on average, younger and had fewer comorbidities compared with patients initiating 1 L NHT. Patients without life-prolonging therapy numerically had the highest proportion of men who were Black (16%), the proportion of Black patients was lower among patients initiating NHTs (15%), chemotherapy (12%), sipuleucel-T (11%), combination & other (10%), and radium-223 (8%) (Table 1). Among patients with baseline NHT use (1,519 patients), 70% received 1 L NHT after mCRPC diagnosis, 7% chemotherapy, 6% combination & other, 2% sipuleucel-T, 1% radium-223, and 13% had no subsequent life-prolonging therapy. Among patients with baseline taxane use (461 patients), 79% received 1 L chemotherapy after mCRPC diagnosis, 7% NHT, 5% combination & other, 2% sipuleucel-T or radium-223, and 7% had no subsequent life-prolonging therapy.

### Lines of treatment

**Fig. 2:**
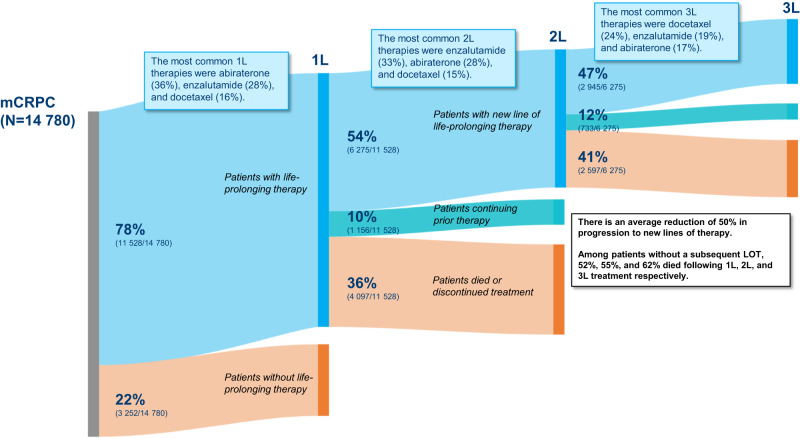
Proportion of patients receiving life-prolonging therapies for mCRPC by LOT. The end of a LOT was defined as a treatment change or at least 90 days without treatment. If the LOT had not ended as of the data cut-off, patients were considered to have treatment ongoing. LOT: line of therapy; 1/2/3 L: 1^st^/2^nd^/3^rd^ LOT.

Among the 78% of patients who initiated 1 L therapy, 54% subsequently received a 2 L therapy, and 47% of those who received 2 L continued to 3 L therapy, reflecting an approximate 50% reduction in treatment across each LOT (Fig. 2). Among patients without a subsequent LOT, 52%, 55%, and 62% died following 1 L, 2 L, and 3 L treatment respectively.

NHTs were the most common therapy class within each of the 3 LOTs studied (Supplemental Fig. S1; Supplemental Table S3). The three most common treatments across all LOTs were abiraterone, enzalutamide, and docetaxel, although ordering varied based on LOT (Fig. 2). The most common therapies were abiraterone (36%), enzalutamide (28%), and docetaxel (16%) for 1 L; enzalutamide (33%), abiraterone (28%), and docetaxel (15%) for 2 L; and docetaxel (24%), enzalutamide (19%), and abiraterone (17%) for 3 L.

Evaluating treatment sequences, the most common 1 L to 2 L regimen was NHT to NHT (33% of all 1 L to 2 L sequences), and NHTs were present as either 1 L or 2 L or both in the five most common treatment patterns (Supplemental Fig. S2). The most common 1 L to 2 L to 3 L regimen was NHT to NHT to chemotherapy (14% of sequences), and NHTs comprised at least two LOTs in the four most common treatment sequences (Supplemental Table S3; Supplemental Fig. S3). For 37% of all mCRPC patients, NHTs were the only life-prolonging therapy they received. For those initiating 1 L treatment with NHTs, only 28% received subsequent treatment with a different class of therapy. Across all LOTs, only 26% of all mCRPC patients and 34% of those treated with life-prolonging therapy received at least one line of chemotherapy.

Median follow-up after mCRPC diagnosis was 17.0 months. Median time on therapy was 4.0 months for 1 L, 3.7 months for 2 L, and 3.0 months for 3 L. Median time on therapy was shortest for sipuleucel-T (1.0 month) followed by chemotherapy (2.4–3.5 months across LOTs), radium-223 (2.8–3.9 months), NHT (3.4–6.3 months) and combination & other therapy (4.0–6.3 months). Similarly, reductions in time to the next LOT were observed in time to the next therapy from 1 L to 2 L (13.7 months), from 2 L to 3 L (10.9 months), and from 3 L to 4 L (8.9 months) (Table 2). Median time to next LOT was shortest for sipuleucel-T (5.2–6.3 months across LOTs) followed by chemotherapy (8.1–10.7 months), radium-223 (9.3–10.8 months), combination & other therapy (9.7–13.2 months), and NHT (10.2–16.3 months).

**Table 2:** Duration of LOTs by treatment class among mCRPC patients^1,2^.

Outcome	All treatments	NHT	Chemotherapy	Sipuleucel-T	Radium-223	Combination & Other
**LOT #1**						
*N* (%)	11528 (100%)	7556 (66%)	2005 (17%)	1189 (10%)	317 (3%)	461 (4%)
Months on treatment, median [IQR]	4.0 [1.7–9.1]	6.3 [2.6–11.9]	3.5 [2.1–3.8]	1.0 [1.0–1.0]	3.9 [1.9–4.9]	6.3 [3.5–11.1]
Months to LOT #2 or end of follow-up, median [95% CI]	13.7 [13.2–14.0]	16.3 [15.8–16.9]	10.7 [10.1–11.2]	6.3 [5.7–6.9]	10.8 [8.6–13.6]	13.2 [10.5–14.8]
Received LOT #2, n (%)	6275 (54%)	3566 (47%)	1394 (70%)	906 (76%)	157 (50%)	252 (55%)
**LOT #2**						
*N* (%)	6275 (100%)	3912 (62%)	1228 (20%)	186 (3%)	303 (5%)	646 (10%)
Months on treatment, median [IQR]	3.7 [1.9–7.1]	4.3 [2.0–8.8]	2.8 [1.4–4.3]	1.0 [0.5–1.0]	3.4 [1.0–4.7]	5.0 [3.0–9.0]
Months to LOT #3 or end of follow-up, median [95% CI]	10.9 [10.5–11.6]	12.8 [12.1–13.5]	8.5 [8.0–9.3]	5.2 [4.4–8.2]	9.3 [7.8–10.2]	11.5 [10.5–13.2]
Received LOT #3, n (%)	2945 (47%)	1793 (46%)	576 (47%)	122 (66%)	145 (48%)	309 (48%)
**LOT #3**						
*N* (%)	2945 (100%)	1130 (38%)	1092 (37%)	42 (1%)	258 (9%)	423 (14%)
Months on treatment, median [IQR]	3.0 [1.4–5.2]	3.4 [1.9–6.3]	2.4 [1.1–4.2]	*(redacted)*	2.8 [1.0–4.7]	4.0 [2.5–6.7]
Months to LOT #4 or end of follow-up, median [95% CI]	8.9 [8.5–9.3]	10.2 [9.1–11.4]	8.1 [7.3–8.6]	*(redacted)*	9.4 [7.6–11.0]	9.7 [8.4–10.6]
Received LOT #4, n (%)	1361 (46%)	510 (45%)	512 (47%)	27 (64%)	114 (44%)	198 (47%)

*Medians for cell sizes <50 are redacted; *CI* Confidence interval, *IQR* Interquartile range, *LOT* Line of therapy, *NHT* Novel hormonal therapy

1. Months on treatment for each LOT was measured as time from the start of the LOT to the end of treatment (earliest of last treatment, start of the next LOT, death, or data cut-off)

2. Months to the next LOT was calculated as the time from the start of the LOT to the start of the next LOT, estimated from Kaplan-Meier survival analyses censoring for death or data cut-off.

### Survival

**Fig. 3:**
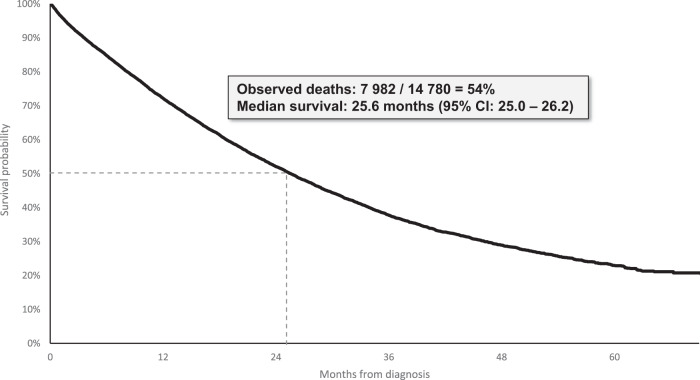
Overall survival from mCRPC diagnosis. CI: Confidence interval.

Overall mortality in the cohort was 54%. Median survival from mCRPC diagnosis was 25.6 months (95% confidence interval [CI] 25.0–26.2 months) (Fig. 3). Median survival from treatment initiation was 23.4 months (95% CI: 22.8–24.1 months) (Supplemental Fig. S4).

## Discussion

In this contemporary national evaluation of Medicare patients with evidence of mCRPC, only 78% of patients received any life-prolonging therapy after mCRPC diagnosis, with NHTs as the most common treatment. Over a third (37%) of men received life-prolonging therapy only with NHTs, and multiple LOTs with NHTs were common. As an additional 22% of men did not receive any life-prolonging therapy, less than half of men were treated with two different classes of treatments following mCRPC diagnosis. Following 1 L, approximately half of the patients received 2 L, with mortality being the most common censoring event among those not continuing therapy. The median time from therapy initiation to the next LOT or end of follow-up was 13.7 months from the start of 1 L, 10.9 months from the start of 2 L, and 8.9 months from the start of 3 L. More than half of the patients died during the study period, with a median survival of 25.6 months.

This study complements previously published work evaluating real-world mCRPC treatment patterns and outcomes, extending findings to the older adult population represented by Medicare. As noted by others [1], more than 20% of patients do not begin life-prolonging therapy after mCRPC diagnosis. Advanced age and higher comorbidity burdens in this group suggest that this may reflect consideration of broader goals of care. Similarly, there was an approximately 50% reduction in the per cent of patients receiving each subsequent LOT, with loss driven primarily by intervening mortality. Moses and colleagues [8] did note higher proportions of patients receiving subsequent LOTs than found in the current study in their evaluation of commercial and Medicare claims. This difference likely reflects their inclusion of non-life-prolonging therapies such as leuprolide and bicalutamide in their definition of LOTs, which were not incorporated in this analysis given the focus on life-prolonging therapies.

The high proportion of NHT usage, particularly as the basis of 1 L and 2 L therapies, is reassuring and reflects the latest NCCN Clinical Practice Guidelines in Oncology (NCCN Guidelines®); however, patients receiving multiple LOTs of NHTs is not consistent with guidelines and is a potential cause of concern [14]. In the latest NCCN Guidelines®, published September 2022 (Version 1.2023), preferred regimens for individuals with prior NHT therapy are docetaxel, olaparib, and radium-223 (Category 1 recommendation) [14]. NHTs are included in the *other recommended* regimens group of options for this cohort [14]. The common use of NHTs has also been reported by Malangone-Monaco et al. (2022), George et al. (2020), and Shore et al. (2021), indicating that NHT usage is not limited to a particular demographic or insurance population of mCRPC [1–3]. Similarly, these findings align with previous work documenting median NHT usage of approximately 7 months and longest when used as part of 1 L [1, 2]. The current study’s proportion of 1 L NHT to 2 L NHT use in the Medicare population is also comparable to that reported by Malangone-Monaco et al. in a commercial population, where it was also the most common 1 L to 2 L treatment sequence [3].

The median survival of 25.6 months from mCRPC diagnosis is higher than the 21.2 months reported in a previous study of electronic health records [1]. The slight difference may be explained by differences in settings of care. In the prior study of electronic health records, the authors noted that the majority of patients were treated in a community setting, where patient characteristics and physician practice patterns may differ from those at subspecialty academic centers. Median survival of 23.4 months from treatment initiation is also slightly higher than a 19.4-month estimate using electronic health records, but comparable to survival for patients who received life-prolonging therapies in the same database (23.7 months) [1, 2].

The strengths of this study include its evaluation of a 100% fee-for-service Medicare sample, thereby providing the first comprehensive evaluation of real-world treatment patterns and outcomes among elderly patients in fee-for-service Medicare. Medicare data also provide longer follow-up and more reliable measurement of mortality than is typically found in commercial or open claims databases. Understanding treatment and outcomes in this population can better inform both clinical management and research for the majority of mCRPC patients.

This study has several limitations. First, the algorithm used to identify mCRPC cases [12] has not been evaluated against formal chart review; thus, its accuracy in case identification is unknown. Given high levels of treatment with life-prolonging therapy and similar findings to those in electronic health record studies, errors would most likely lead to diminished sensitivity for mCRPC cases that did not undergo treatment. Second, a lag in the availability of Medicare data limited the current study’s evaluation to 2019 and earlier, thus precluding incorporation of subsequently approved mCRPC therapies including rucaparib, olaparib, and lutetium Lu 177 vipivotide tetraxetan. Third, some of the chemotherapy treatments included in this study lack evidence that they extend survival in mCRPC; however, are referred to as life-prolonging treatments for simplicity. Fourth, the current analysis is based on administrative claims data, which lack potentially important clinical details. Fifth, in accordance with terms of Medicare data use, cells with patient counts less than 11 were redacted. Importantly, overall survival was analyzed descriptively without adjusting for confounding factors or comparison to a non-mCRPC cohort, and thus estimates should not be interpreted as a causal effect of mCRPC on overall survival. Clinical characteristics unobserved in the data likely inform treatment selection and outcomes.

## Conclusion

Most Medicare-insured men with mCRPC did not receive a life-prolonging therapy or had only 1 L therapy after mCRPC diagnosis, with a 50% without further treatment after each line of therapy. NHTs were the most common 1 L and 2 L therapies, and NHT followed by a different NHT was the most common treatment sequence. Further research is needed to understand how treatment patterns change as NHTs and docetaxel are used earlier in the disease continuum and as new therapies are introduced and ultimately to identify optimal treatment sequencing. Nonetheless, the current data suggest a dramatic undertreatment of men with mCRPC.

### Supplementary information

Supplemental Table S1: Additional baseline characteristics by 1L treatment category

Supplemental Table S2: Baseline characteristics by number of lines of therapy

Supplemental Table S3: Top 5 most common treatment sequences among mCRPC patients

Supplemental Figure S1: Distribution of treatments across mCRPC LOTs

Supplemental Figure S2: Top 10 most frequent 1L to 2L sequences

Supplemental Figure S3: Top 10 most frequent 1L to 2L to 3L sequences

Supplemental Figure S4: Overall survival from 1L treatment initiation

## Data Availability

The data that support the findings of this study are available from the U.S. Centers for Medicare & Medicaid Services, but restrictions apply to the availability of these data, which were used under license for the current study, and so are not publicly available.
