# Supplementary material for: Real-world treatment patterns and overall survival among men with Metastatic Castration-Resistant Prostate Cancer (mCRPC) in the US Medicare population
Source: Prostate Cancer Prostatic Dis. 2023 Oct 2;27(2):327–33. doi: 10.1038/s41391-023-00725-8 (PMC11096091; doi:10.1038/s41391-023-00725-8)
Supplement: Supplementary file 1 — Supplemental Table S1: Additional baseline characteristics by 1L treatment category [file 41391_2023_725_MOESM1_ESM.pdf]

Supplemental Table S1: Additional baseline characteristics by 1L treatment category[1,2]

Characteristic	Overall	NHT	Chemotherapy	Sipuleucel-T	Radium-223	Combination & Other	Without life-prolonging treatment
**N (%)**	14 780 (100%)	7 556 (51%)	2 005 (14%)	1 189 (8%)	317 (2%)	461 (3%)	3 252 (22%)
**Index year, n (%)**							
2014	1 313 (9%)	643 (9%)	222 (11%*)	136 (11%*)	(redacted)	25 (5%)	278 (9%)
2015	2 333 (16%)	1 182 (16%)	361 (18%)	179 (15%)	57 (18%)	85 (18%)	469 (14%)
2016	2 683 (18%)	1 218 (16%)	473 (24%*)	238 (20%*)	76 (24%*)	90 (20%)	588 (18%)
2017	3 233 (22%)	1 654 (22%)	454 (23%)	263 (22%)	74 (23%)	116 (25%)	672 (21%)
2018	3 659 (25%)	2 045 (27%)	355 (18%*)	274 (23%*)	80 (25%)	103 (22%)	802 (25%*)
2019	1 559 (11%)	814 (11%)	140 (7%*)	99 (8%*)	(redacted)	42 (9%)	443 (14%*)
**CCI components, n (%)**							
Acute myocardial infarction	459 (3%)	250 (3%)	51 (3%)	20 (2%*)	(redacted)	14 (3%)	114 (4%)
History myocardial infarction	1 093 (7%)	564 (7%)	129 (6%)	55 (5%*)	28 (9%)	30 (7%)	287 (9%)
Cerebrovascular disease	2 254 (15%)	1 177 (16%)	235 (12%*)	143 (12%*)	45 (14%)	55 (12%)	599 (18%*)
Congestive heart failure	2 530 (17%)	1 360 (18%)	240 (12%*)	119 (10%*)	52 (16%)	64 (14%)	695 (21%*)
Chronic pulmonary disease	3 383 (23%)	1 719 (23%)	435 (22%)	195 (16%*)	81 (26%)	100 (22%)	853 (26%*)
Dementia	787 (5%)	399 (5%)	27 (1%*)	23 (2%*)	13 (4%)	16 (3%)	309 (10%*)
Diabetes w/out chronic complications	5 009 (34%)	2 563 (34%)	637 (32%)	365 (31%)	106 (33%)	152 (33%)	1 186 (36%)
Diabetes w/chronic complications	2 218 (15%)	1 187 (16%)	234 (12%*)	133 (11%*)	44 (14%)	56 (12%)	564 (17%)
Hemiplegia or paraplegia	249 (2%)	140 (2%)	32 (2%)	(redacted)	(redacted)	(redacted)	59 (2%)
HIV/AIDS	33 (0%)	15 (0%)	(redacted)	(redacted)	(redacted)	(redacted)	(redacted)
Mild liver disease	1 843 (12%)	909 (12%)	325 (16%*)	149 (13%)	39 (12%)	76 (16%*)	345 (11%)
Moderate/Severe liver disease	76 (1%)	37 (0%)	(redacted)	(redacted)	(redacted)	(redacted)	19 (1%)
Peptic ulcer disease	302 (2%)	172 (2%)	34 (2%)	16 (1%)	(redacted)	(redacted)	62 (2%)
Peripheral vascular disease	3 649 (25%)	1 846 (24%)	432 (22%*)	233 (20%*)	79 (25%)	115 (25%)	944 (29%*)
Renal failure	3 309 (22%)	1 785 (24%)	349 (17%*)	220 (19%*)	50 (16%*)	96 (21%)	809 (25%)
Rheumatic disease	374 (3%)	192 (3%)	50 (2%)	18 (2%)	(redacted)	(redacted)	95 (3%)

*P ≤ 0.01 vs NHT treatment; Abbreviations: CCI: Charlson Comorbidity Index; NHT: Novel hormonal therapy; HIV/AIDS: Human immunodeficiency virus / acquired immunodeficiency syndrome

1. Individuals are categorized into treatment categories based on their 1L treatment
2. Counts and proportions for cell sizes <11 are redacted.
