# Supplementary material for: Real-world treatment patterns and overall survival among men with Metastatic Castration-Resistant Prostate Cancer (mCRPC) in the US Medicare population
Source: Prostate Cancer Prostatic Dis. 2023 Oct 2;27(2):327–33. doi: 10.1038/s41391-023-00725-8 (PMC11096091; doi:10.1038/s41391-023-00725-8)
Supplement: Supplementary file 2 — Supplemental Table S2: Baseline characteristics by number of lines of therapy [file 41391_2023_725_MOESM2_ESM.pdf]

Supplemental Table S2: Baseline characteristics by number of lines of therapy[1,2]

Characteristic	Overall	Without life-prolonging treatment	1 line of life-prolonging treatment	2 lines of life-prolonging treatment	3+ lines of life-prolonging treatment
**N (%)**	14 780 (100%)	3 252 (22%)	5 253 (36%)	3 330 (23%)	2 945 (20%)
**Demographics**					
**Age in years, mean (SD)**	76.1 (7.3)	78.1* (7.9)	77.8 (7.4)	75.6* (6.9)	73.4* (5.7)
**Race, n (%)**					
Non-Hispanic White	11 033 (75%)	2 393 (74%)	3 837 (73%)	2 472 (74%)	2 331 (79%*)
Black or African American	2 079 (14%)	513 (16%)	777 (15%)	472 (14%)	317 (11%*)
Asian/Pacific Islander	381 (3%)	79 (2%)	133 (3%)	96 (3%)	73 (2%)
Hispanic	824 (6%)	181 (6%)	325 (6%)	189 (6%)	129 (4%*)
Other/Unknown[3]	463 (3%)	86 (3%)	181 (3%)	101 (3%)	95 (3%)
**Geographic region, n (%)**					
Northeast	2 709 (18%)	590 (18%)	1 009 (19%)	596 (18%)	514 (17%)
Midwest	3 857 (26%)	894 (27%)	1 335 (25%)	848 (25%)	780 (26%)
South	5 287 (36%)	1 229 (38%*)	1 821 (35%)	1 220 (37%)	1 017 (35%)
West	2 927 (20%)	539 (17%*)	1 088 (21%)	666 (20%)	634 (22%)
**Baseline treatment**					
Baseline NHT use	1 519 (10%)	200 (6%*)	680 (13%)	341 (10%*)	298 (10%*)
Baseline taxane use	461 (3%)	33 (1%*)	130 (2%)	146 (4%*)	152 (5%*)
**CCI, mean (SD)**	2.1 (2.0)	2.4 (2.2)	2.3 (2.1)	2.0* (1.9)	1.7* (1.7)

*P ≤ 0.01 vs 1 line of treatment; Abbreviations: CCI: Charlson Comorbidity Index; NHT: Novel hormonal therapy; SD, Standard deviation

1. Individuals are categorized into categories based on the number of lines of therapy observed.
2. Baseline characteristics are evaluated prior to 1st line treatment initiation.
3. Other/unknown includes 60% unknown, 30% other, and 11% American Indian / Alaska Native.
