# Supplementary material for: Real-world treatment patterns and overall survival among men with Metastatic Castration-Resistant Prostate Cancer (mCRPC) in the US Medicare population
Source: Prostate Cancer Prostatic Dis. 2023 Oct 2;27(2):327–33. doi: 10.1038/s41391-023-00725-8 (PMC11096091; doi:10.1038/s41391-023-00725-8)
Supplement: Supplementary file 3 — Supplemental Table S3: Top 5 most common treatment sequences among mCRPC patients [file 41391_2023_725_MOESM3_ESM.pdf]

Supplemental Table S3:
Top 5 most common treatment sequences among mCRPC patients

First line (1L)	Second Line (2L)	Third Line (3L)	N (%)
**1L → 2L treatment sequence**			**6 275 (100%)**
NHT	NHT		2 091 (33%)
Chemotherapy	NHT		898 (14%)
NHT	Chemotherapy		794 (13%)
Sipuleucel-T	NHT		730 (12%)
NHT	Combination & Other		414 (7%)
**1L → 2L → 3L treatment sequence**			**2 945 (100%)**
NHT	NHT	Chemotherapy	408 (14%)
Chemotherapy	NHT	NHT	216 (7%)
Sipuleucel-T	NHT	NHT	207 (7%)
NHT	NHT	NHT	194 (7%)
Chemotherapy	NHT	Chemotherapy	156 (5%)

Abbreviations: mCRPC: Metastatic castration-resistant prostate cancer; NHT: Novel hormone therapy 1/2/3L: 1st/2nd/3rd Line of therapy
