# Supplementary figures and images for: Real-world treatment patterns and overall survival among men with Metastatic Castration-Resistant Prostate Cancer (mCRPC) in the US Medicare population
Source: Prostate Cancer Prostatic Dis. 2023 Oct 2;27(2):327–33. doi: 10.1038/s41391-023-00725-8 (PMC11096091; doi:10.1038/s41391-023-00725-8)

# Supplemental Figure S1: Distribution of treatments across mCRPC LOTs

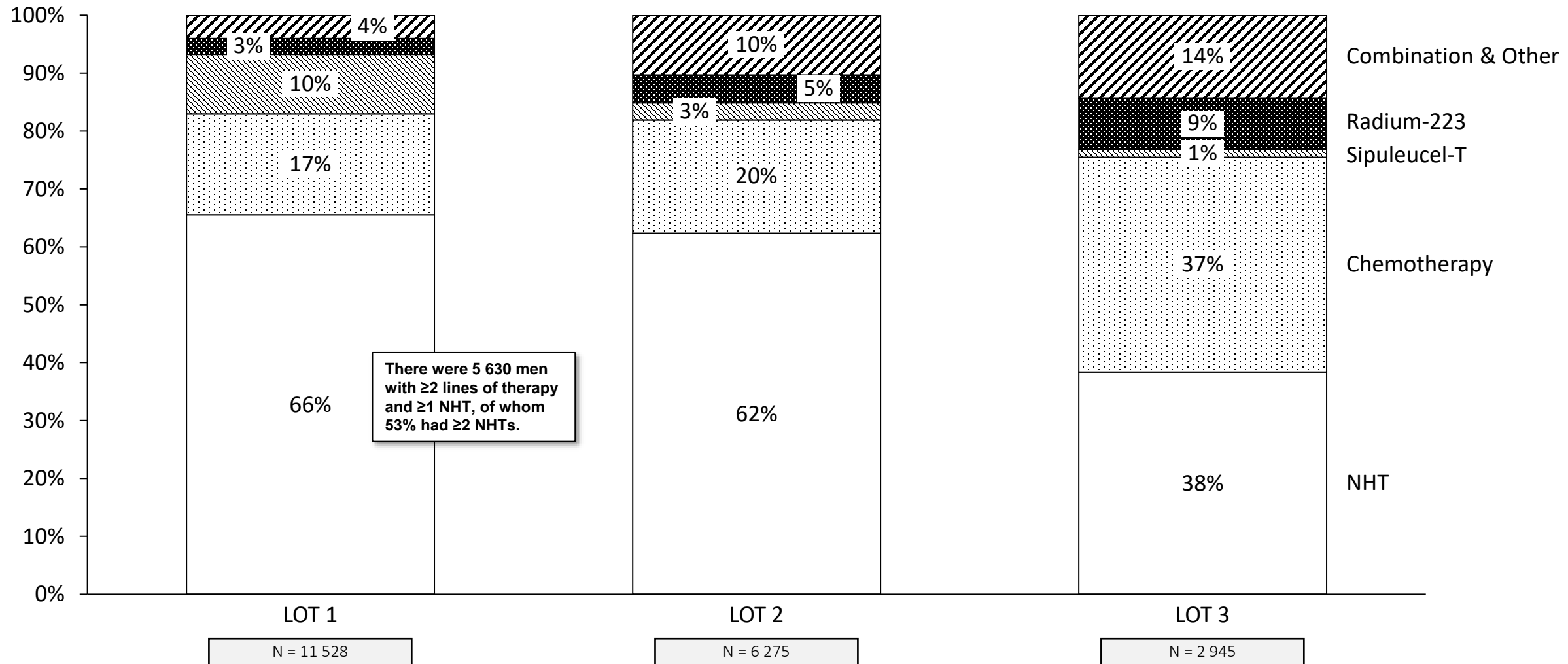

Supplement: Supplementary file 4 — Supplemental Figure S1: Distribution of treatments across mCRPC LOTs [file 41391_2023_725_MOESM4_ESM.pdf]

# Supplemental Figure S2. Top 10 most frequent 1L to 2L sequences

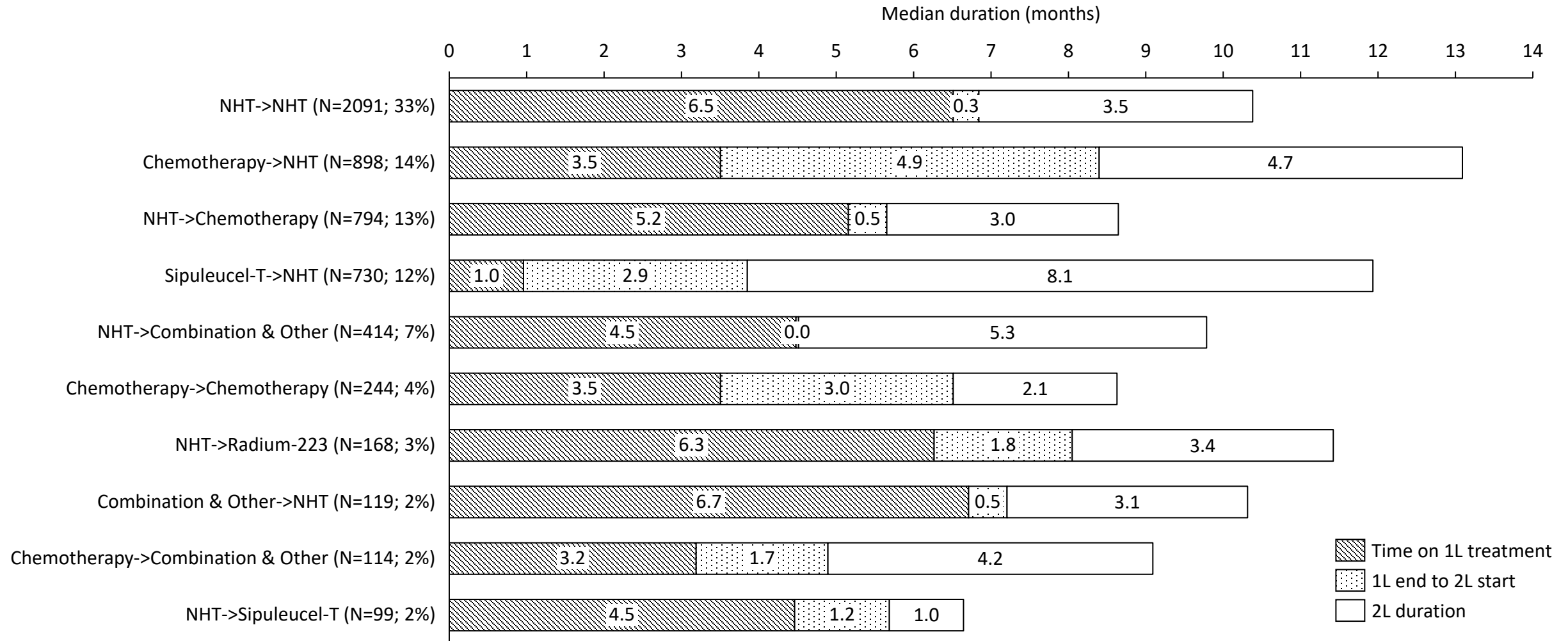

Supplement: Supplementary file 5 — Supplemental Figure S2: Top 10 most frequent 1L to 2L sequences [file 41391_2023_725_MOESM5_ESM.pdf]

# Supplemental Figure S3. Top 10 most frequent 1L to 2L to 3L sequences

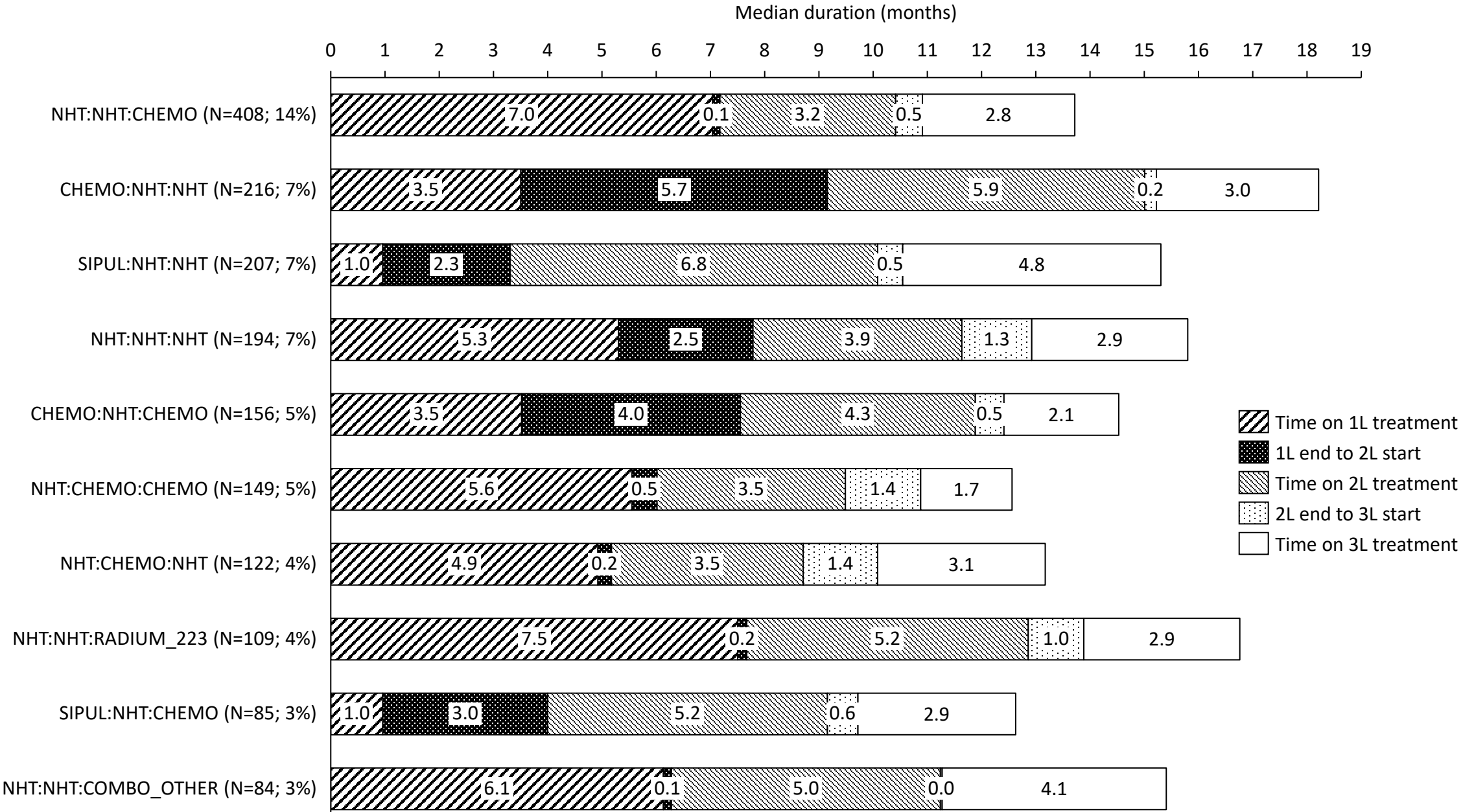

Supplement: Supplementary file 6 — Supplemental Figure S3: Top 10 most frequent 1L to 2L to 3L sequences [file 41391_2023_725_MOESM6_ESM.pdf]
