# Supplementary material for: Real-world treatment patterns and overall survival among men with Metastatic Castration-Resistant Prostate Cancer (mCRPC) in the US Medicare population
Source: Prostate Cancer Prostatic Dis. 2023 Oct 2;27(2):327–33. doi: 10.1038/s41391-023-00725-8 (PMC11096091; doi:10.1038/s41391-023-00725-8)
Supplement: Supplementary file 7 — Supplemental Figure S4: Overall survival from 1L treatment initiation [file 41391_2023_725_MOESM7_ESM.pdf]

Supplemental Figure S4:
Overall survival from 1L treatment initiation

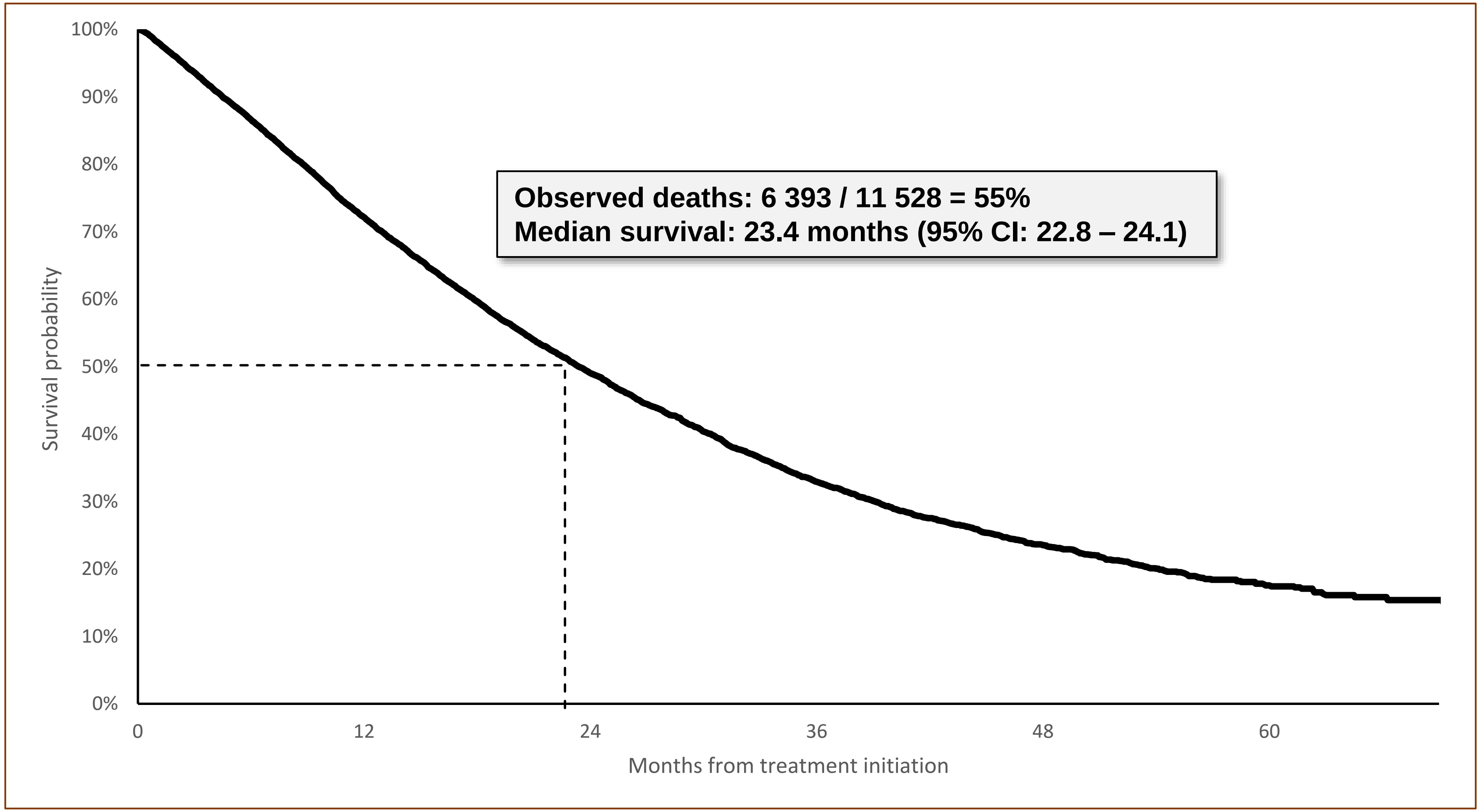

Abbreviations: CI: Confidence interval
